# Recent Developments in Fluorescent Materials for Heavy Metal Ions Analysis From the Perspective of Forensic Chemistry

**DOI:** 10.3389/fchem.2020.593291

**Published:** 2020-11-10

**Authors:** Jie Lian, Qiang Xu, Yipeng Wang, Fanda Meng

**Affiliations:** ^1^College of Criminal Investigation, People's Public Security University of China, Beijing, China; ^2^Institute of Basic Medicine, Shandong First Medical University & Shandong Academy of Medical Sciences, Shandong, China

**Keywords:** sensor, heavy metal ion, fluorescent detection, forensic chemistry, fluorescent material

## Abstract

Forensic chemistry deals with the analysis of various types of physical evidences related to crime, corresponding to the detection of target substances or elements in complex matrices. There is a vital need for highly selective, rapid, and sensitive biosensing technologies in heavy metal ions analysis especially those from living persons, autopsy, food, water, soil, and other identified substances at very preliminary stages. Fluorescent materials-based method for heavy metal ions detection is one of the most important analytical methods, resulting in the ability to measure analytes in complex matrices with unsurpassed selectivity and sensitivity. In this mini review, different fluorescent materials-based analytical methods aiming at several heavy metal ions detection are exclusively reviewed through a comprehensive literature survey. In addition, current challenges to achieve integrated evidence analysis process are briefly discussed to provide an outlook for heavy metal ions detection based on fluorescent analytical methods in the forensic chemistry field.

## Introduction

Nowadays, the proper application of science is of utmost importance in solving crimes all around the world. Forensic chemistry is one of the powerful tools to connect the suspect with the crime scene or the victim by matching physical evidence from the crime scene or victim with evidence found on or about the person accused of the crime. Forensic chemistry deals with the identification and analysis of chemical components of evidence found at the scene of a crime in order to link the evidence to the perpetrator of the crime. One of the fundamental tasks in forensic chemistry is to determine the presence and analyze the concentrations of particular components in various types of physical evidences taken from the crime scene and living persons and even collected during autopsy, as well as in food, water, sewage, soil, human body fluids, and other identified substances (Kloosterman et al., 2015; Pereira de Oliveira et al., 2018; De Kinder and Pirée, 2020). Evidence analysis task always faces the challenges of limited samples, complex matrices, and ambiguous interpretations. The analytical approaches in this process are making the findings acceptable by the judiciary based on different technologies in chemistry.

Heavy metal ions are one of the most important parameters in the toxicology and trace evidence examination, covering suicide, homicide, illegal drug, explosion, robbery, and terrorist cases (Lachas et al., 2000; Ababneh and Al-Momani, 2018; Verma, 2018; Santos et al., 2019; Brown et al., 2020; Yusoff et al., 2020). In day-to-day life, human beings are encountered with useful and useless chemical substances made of heavy metal elements. Heavy metal elements are considered as inorganic irritant poisons, endangering to human health and depending upon the chemical nature, administration route, and amount of the element (Amarnath and Shukla, 2014). Heavy metals include lead, mercury, copper, chromium, thallium, cadmium, copper, arsenic, iron, silver, and the platinum group elements and occur naturally in ecosystems, leading to unusual symptoms and even death from acute, chronic, or acute-on-chronic exposure. Nickel, aluminum, iron, tin, copper, strontium, zinc, iron, and titanium are the key elements in the analysis of gunshot residue, paint, and glass evidences. There are a wide array of instrumental methods allowing identification and determination of various metal elements in different evidences, such as atomic absorption spectroscopy, neutron activation analysis, X-ray fluorescence spectroscopy, ion chromatography, mass spectroscopy, and other spectroscopy coupled with mass spectroscopy (Ulrich et al., 2004; Verma, 2018; Sliwińska et al., 2019). Compared with these traditional analytical methods, fluorescence spectroscopy is more suitable for trace metal ions analysis in complex matrices because of its short response time, simplicity, high selectivity, and sensitivity (Fu et al., 2019; Zhang et al., 2019; Niu et al., 2020; Zang et al., 2020).

This mini review intends to provide an overview of the recent progress of fluorescent materials for heavy metal ions detection in different aqueous matrices or real samples mainly in the recent 5 years, considering the reagents' safety, selectivity, and sensitivity. Finally, the existing challenges and future perspectives for the application of fluorescent materials-based analytical methods in forensic chemistry are briefly discussed.

## Fluorescent Molecules for Heavy Metal Ions Detection

Small-molecule-based fluorescence detection methods are preferable approaches to measure heavy metal ions since the change in fluorescence caused by coordination is rapid, non-destructive, selective, highly sensitive, and suitable for screening applications. These methods focus on the design and synthesis of coordination ligand-contained fluorophores and the binding process for metal ions sensing in solution. Fluorescent sensors for Hg^2+^, Cu^2+^, Zn^2+^, Cd^2+^, Fe^3+^, Ni^2+^, Cd^2+^, and Cr^2+^ are developed by conjunction with suitable probes containing the core fluorophores, such as rhodamine, pyrene, anthracene, naphthalimide, aminoquioline, bithiophene, and coumarin (Saleem et al., 2017; Sivaraman et al., 2018; Bai et al., 2019, 2020).

### Fluorescent Sensors Based on Rhodamine B-Related Molecules

**Figure 1:**
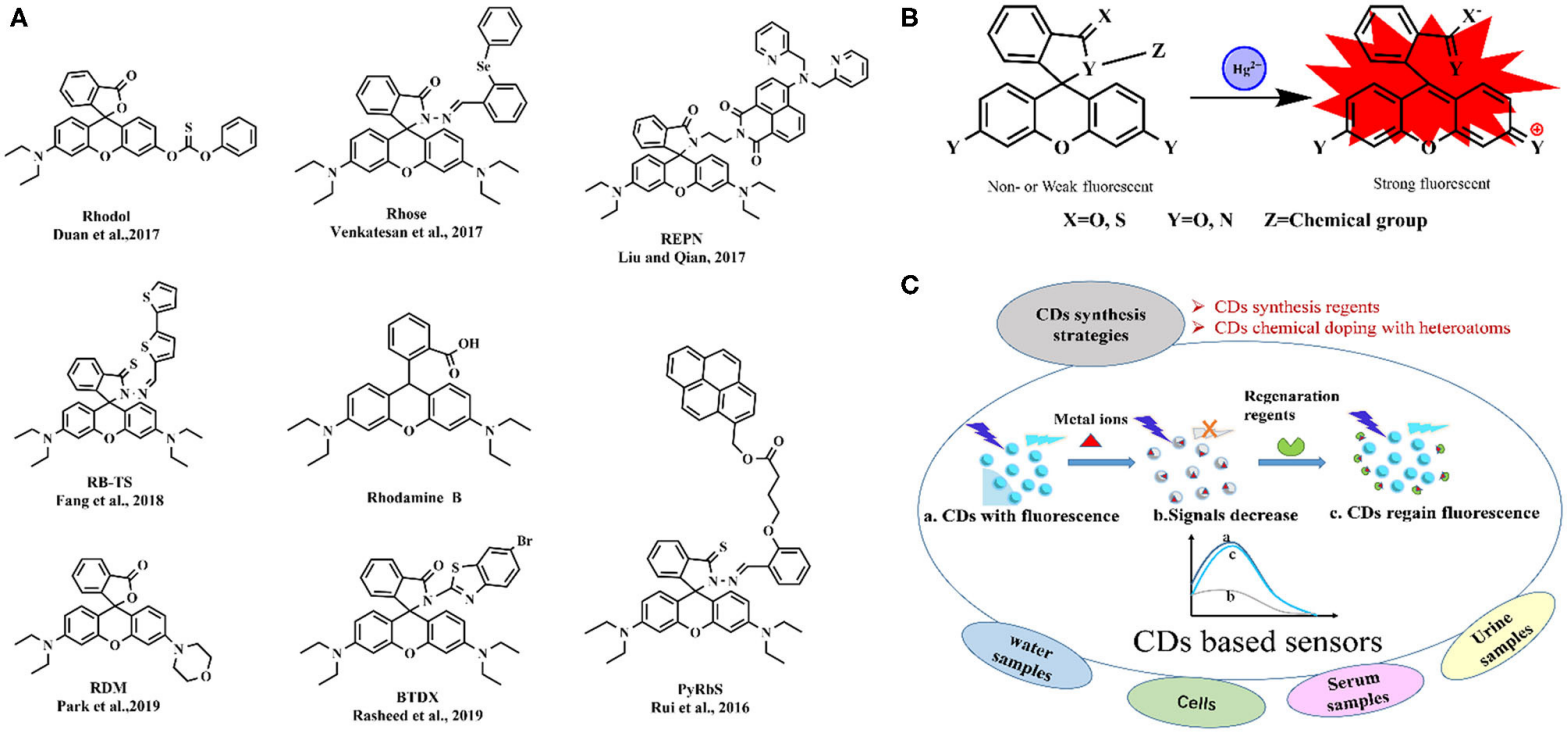
Fluorescence molecular structures based on rhodamine B for Hg^2+^ biosensing **(A)**, the proposed mechanism for sensing Hg^2+^ by rhodamine B-related molecules **(B)**, and schematic diagram of CDs-based fluorescence research for metal ions detection **(C)**. PyRbS, spirolactone rhodamine-pyrene derivative; Rhose, rhodamine-diphenylselenium derivative; REPN, rhodamine-naphthalimide derivative; Rhodol, rhodol containing carbonothioate derivative; RB-TS, thiooxo-rhodamine B derivative; RDM, rhodamine-morpholine derivative; BTDX, thiooxo-rhodamine-bithiophene derivative.

Rhodamine B is widely used in heavy metal ions detection with high stability, large molar extinction coefficient, and high fluorescence quantum yield. Many molecular structures (Figure 1A) based on rhodamine B are designed and synthesized for more sensitive and more adapted to the sample matrix for Hg^2+^ recognition (Rui et al., 2016; Duan et al., 2017; Liu and Qian, 2017; Venkatesan et al., 2017; Fang et al., 2018; Park et al., 2019; Rasheed et al., 2019), and the proposed mechanism of Hg^2+^ biosensing is in Figure 1B. A rhodamine B-derived Schiff base has been designed for the combined detection of Cu^2+^, Al^3+^, and Fe^3+^, and its sensing behavior toward various metal ions was investigated in semi-aqueous media (Gupta et al., 2016). The sensor showed significant fluorescence response accompanied by a color change from colorless to pink emission to Fe^3+^ over a wide range of metal ions, while just colorimetric response toward Cu^2+^ and Al^3+^. Park et al. (2019) fabricated the paper test strips based on rhodamine-morpholine probe for Hg^2+^ detection, a limit of detection (LOD) of 4.8 μM within 1–2 min treatment. Liu and Qian (2017) produced a fluorescent sensor based on the naphthalimide-rhodamine dye encapsulated in silica nanoparticles to improve its water solubility and biocompatibility, and the ratiometric fluorescent sensor realized quantitative detection of Hg^2+^ with a LOD of 2.72 μM within <2 s in complex sample analysis and bioapplications. Another rhodamine-based sensor with diphenylselenium was developed for highly selective fluorescence detection of Hg^2+^ with a LOD of 12 nM (Venkatesan et al., 2017). Duan et al. (2017) chose rhodol dye (a hybrid structure of rhodamine and fluorescein) containing carbonothioate with high affinity of sulfide moiety to Hg^2+^ and achieved high selectivity and ultrasensitivity for Hg^2+^ in aqueous solution with a LOD of 1.4 nM, which was successfully used in four real water samples and cell bioimaging. Rhodamine-related molecules can also detect other metal ions. A fluorescent sensor based on the product of rhodamine B hydrazide and p-chlorobenzaldehyde exhibited great selectivity and specificity toward Cr^3+^ and Fe^3+^ ions over other tested metal ions with a LOD of 8.64 and 10.5 μM for Cr^3+^ and Fe^3+^, respectively. And it could be reversible with the addition of sodium-EDTA (Sirajuddin and Shkir, 2017). A new rhodamine B hydrazone derivative was synthesized and showed selective response to Fe^3+^ and Cu^2+^ ions at 552 and 545 nm, respectively, showing the LOD of 4.63 nM (Fe^3+^) and 526.4 nM (Cu^2+^) (Wang et al., 2017). A phenothiazine-rhodamine-based fluorogenic sensor was developed for Zn^2+^ with a LOD of 289 μM and demonstrated the feasibility of bioimaging use in HeLa cells (Karmegam and Subramanian, 2020).

### Fluorescent Sensors Based on Other Fluorescent Molecules

Other fluorescent molecules have been also synthesized to achieve high sensitivity and improved water solubility for biosensing and bioimaging. An 8-aminoquioline-based fluorescent sensor was synthesized to detect Zn^2+^ with a LOD of 2.15 nM, efficiently monitoring Zn^2+^ changes in a broad pH range from 4.0 to 11.0 without interference from other metal ions and was biocompatible to monitor Zn^2+^ in living cells (Chen et al., 2020b). A highly sensitive fluorescent sensor based on tetraphenylethene (TPE)-functionalized quinolinium salts with aggregation-induced emission (AIE) characteristics was developed for the detection of I^−^ and Hg^2+^, with a fluorescence “turn-on” signal for Hg^2+^ (LOD of 71.8 nM) and “turn-off” for I^−^ (LOD of 22.6 nM). The sensor was successfully used in real samples, such as running water and urine (Zhang et al., 2016). Inspired by the unique AIE feature, another novel TPE derivative containing sulfonic groups for water solubility modulation and carboxyl dithioacetals for Hg^2+^ sensing was grafted on electrospun fiber to fabricate Hg^2+^ test papers with the LOD of 20 nM in 30 min (Zhao et al., 2020). Bithiophene-based water-soluble fluorescent probe was developed for highly sensitive (19 nM) and ultrarapid (within 20 s) detection of Hg^2+^, showing high application performances for Hg^2+^ detection in real water, seafood, urine, and live cells, as well as a powerful molecular tool for the fluorescence bioimaging (Li et al., 2020a).

### A Series Research of Heavy Metal Detection for Real Samples

Talio et al. have developed a series of fluorescent methods with sample preparation strategies for heavy metal ions analysis in real samples compared with the results of inductively coupled plasma-mass spectrometry (ICP-MS), which is the standard method for element quantification in forensic chemistry nowadays. Pb^2+^ in e-cigarette refill liquids were analyzed using rhodamine B as the fluorophore by new solid surface fluorescence methodology. The e-cigarette sample underwent the pre-concentration step based on the formation of coacervate phase using the cationic surfactant cetyltrimet hylammonium bromide and potassium iodine, and then rhodamine B was added to the collected coacervate phase on a filter paper disk to detect Pb^2+^ (Talio et al., 2015). A novel fluorescent application for Ni^2+^ and Cd^2+^ quantification was studied using the fluorophore eosin in varied tobacco samples, such as refill solutions for e-cigarettes, snuff used in narguille, and traditional tobacco, and the sample preparation was interesting for two aimed ions through the chemofiltration on nylon membrane employing eosin and carbon nanotubes solution. Ni^2+^ was selectively retained on the solid support, and Cd^2+^ remained in the filtrate liquid. A spectrofluorimetric determination of both metals was carried out on the solid support (λ_em_ = 545 nm for Ni^2+^) and the filtered aqueous solution (λ_em_ = 565 nm for Cd^2+^) with a LOD of 0.019 and 0.041 μg L^−1^, respectively (Talio et al., 2017). Talio's group also tested Pb^2+^ in leachate of tobacco products and e-cigarettes refill solutions using the fluorophore 8-hydroxyquinoline and o-phenanthroline with a LOD of 0.42 μg L^−1^ (Carolina et al., 2019). The similar methodology was also applied for Pb^2+^ determination in honey (Talio et al., 2019). The aforementioned researches include the sample preconcentration that is extremely important to the analysis of real complex matrices, and the sample preparation is one of the challenging tasks for forensic chemists when using a new technology to forensic chemistry.

## Fluorescent Nanomaterials for Heavy Metal Ions Detection

Nanomaterials display unique optical, electrical, and catalytic properties with high surface reactivity and strong adsorption capacity, and fluorescent nanomaterials-based sensors for heavy metal ions analysis have been developed with remarkable sensitivity. The fluorescent nanosensor is composed of a fluorescent part generating monitorable signals and a receptor part for recognizing particular ions, and fluorescent nanomaterials may play part or both of the two roles. Carbon nanomaterials (such as carbon dots), metal nanomaterials (Choi et al., 2009), quantum dots (QDs), and metal oxide nanomaterials, as alternative or performance improver to fluorescent molecules-based fluorophores, are commonly used. Among the mentioned fluorescent nanomaterials, carbon dots (CDs) have gained considerable interest from the research community with the distinctive advantage of photoluminescence, robust chemical inertness, and excellent biocompatibility, providing a versatile alternative to the currently available systems. Considering the safety of environment and operators, CDs with environmentally friendly synthesis method and good biocompatibility are discussed.

CDs were revealed to be useful as selective sensors for the detection of Ni^2+^, Cr^3+^, Cr^4+^, Pb^2+^, Hg^2+^, and Cu^2+^ (Chini et al., 2019; Khan et al., 2019; Liu et al., 2019; Chaudhary et al., 2020; Singh et al., 2020). Most of the reports focus on heavy metal ions sensing with CDs exhibiting a single fluorescence band that is sensitive to one metal cation only. He et al. found CDs to be effectively quenched by Hg^2+^, while CDs/Hg^2+^ complex could be separated after the addition of I^−^. A fluorescence assay for the detection of Hg^2+^ and I^−^ was developed in real lake water and urine of cattle (He et al., 2016). The concentration of Pb^2+^ was determined based on water-soluble CDs prepared from chocolate, resulting in a LOD as low as 12.7 nM. The proposed method was validated with five real water samples with good spiked recoveries (Liu et al., 2016).

The synthesis strategies of CDs and fluorescent nanosensors used in different matrices have been explored (Figure 1B). Physicochemical and photochemical properties of CDs can be effectively modulated by chemical doping with heteroatoms. Highly blue luminescent nitrogen-doped CDs (N-CDs) with a fluorescence quantum yield of 42.3% were demonstrated as an effective fluorescent sensor for label-free and selective recognition of Fe^3+^ with a LOD of 13.6 nM and imaging of Fe^3+^ in living cells due to Fe^3+^-quenched fluorescence (Han et al., 2016). Nitrogen-doped carbon quantum dots (N-CDs), with a fluorescence quantum yield of 42.2%, prepared using tartaric acid, citric acid, and ethanediamine as the precursors were applied for Hg^2+^ quantification with a LOD of 83.5 nM (Huang et al., 2017). N-CDs prepared by rice residue and glycine were applied as fluorescent sensor to selectively detect Fe^3+^ with a LOD of 746.2 nM, and the results in real water samples were in good agreement with standard ultraviolet–visible method (Qi et al., 2019). Nitrogen and sulfur co-doped CDs (N,S-CDs) with a high quantum yield of 69%, were applied for a highly sensitive and selective determination of Fe^3+^ with a LOD of 14 nM (Qu et al., 2019). Nitrogen and phosphorus co-doped CDs (N,P-CDs) were synthesized and exhibited a strong blue emission and a sensitive response to Fe^3+^ with a LOD of 1.8 nM and showed selective Fe^3+^ detection in living cells. Shi et al. (2016) applied the N,P-CDs for label-free detection of Fe^3+^ in human serum and intracellular fluorescence imaging. B,N,S-co-doped CDs (BNS-CDs) based fluorescent nanosensor with a LOD of 90 nM was successfully applied for efficient detection of Fe^3+^ in human urine and serum samples (Liu et al., 2017).

Fluorescent nanosensor can improve the detection performance by combining with different nanomaterials. A dual-QDs fluorescent sensor selectively detected Ag^+^ contamination in real sample (Chen et al., 2020a). Wang et al. developed an effective fluorescence nanosensor for selective detection of Cu^2+^ by covalently connecting the carboxyl-modified red fluorescent CdTe QDs to the amino-functionalized CDs. The sensor exhibited dual-emission peaks at 437 and 654 nm under a single excitation wavelength of 340 nm, with the red fluorescence for selective recognition of Cu^2+^ and the blue fluorescence as the internal reference. The LOD of this highly sensitive ratiometric sensor is as low as 0.36 nM, and a paper-based sensor has been prepared by printing CDs-QDs probe on a microporous membrane, showing great potential application for on-site screening of Cu^2+^ in real samples (Wang et al., 2016). A novel fluorescent sensor was fabricated through the electrostatic attraction between positively charged N-CDs and negatively charged gold nanoclusters (AuNCs), showing dual-emission peaks at 440 and 565 nm under a single excitation wavelength of 380 nm. Fluorescent ratiometric changes were used for selective and sensitive sensing of Pb^2+^ and Cu^2+^ with the stable blue fluorescence of N-CDs, as the red fluorescence of AuNCs enhanced by Pb^2+^ and quenched by Cu^2+^ (Wang et al., 2019).

## Functional Fluorescent Deoxynucleic Acid for Heavy Metal Ions Detection

Functional deoxynucleic acid has been devoted to developing accurate and selective approaches for heavy metal ions detection, such as single-stranded DNA (ssDNA), DNAzymes, and G-quadruplexes, and the sensitivity can be improved by employing fluorescent molecules and nanomaterials including metal nanoparticles, QDs, and carbon nanomaterials. DNA-related methods have ultrasensitivity and biocompatibility, but researches are limited to several heavy ions due to the complex system components, proper reaction condition requirements, and unique design of DNA molecules.

**Figure 2:**
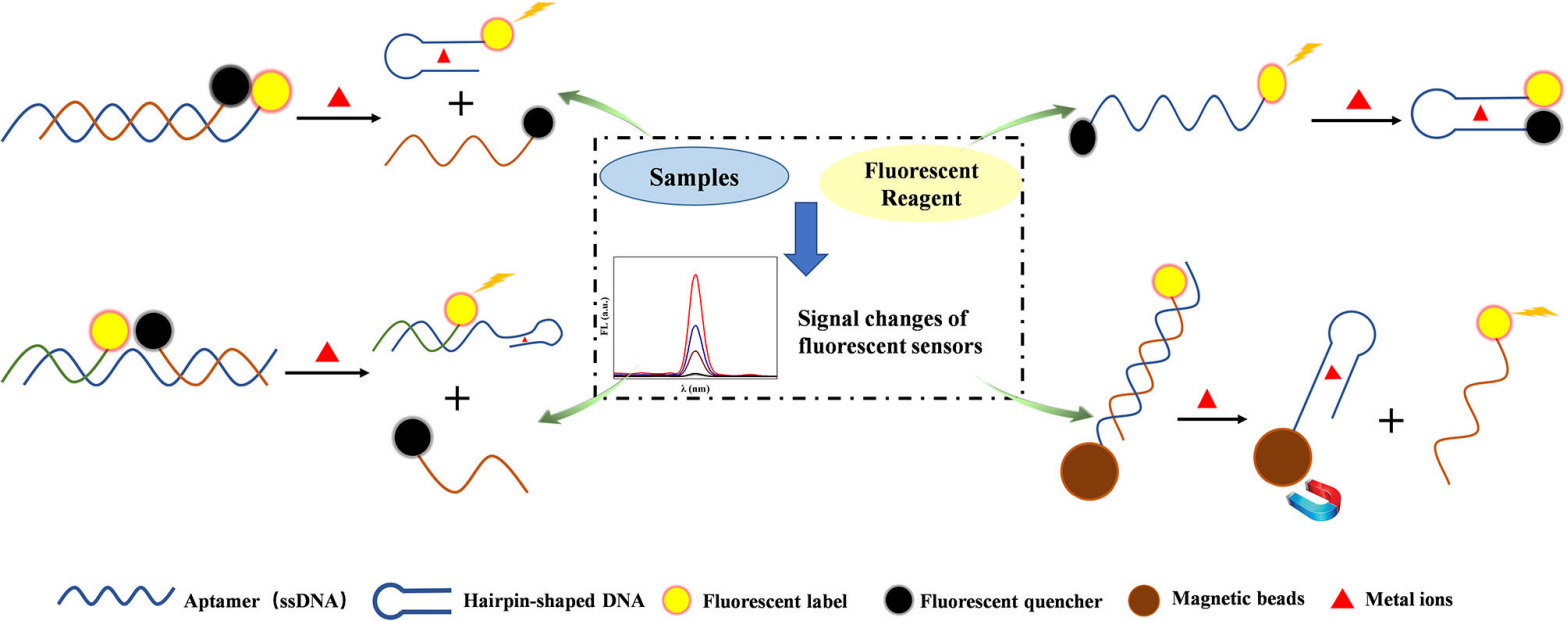
Schematic diagram of several common strategies for aptamer-based fluorescence detection of heavy metal ions. The aptamer can form hairpin structure owing to base pairing with the presence of particular metal ions, and different strategies for metal ions detection are designed based on the structure changes and related fluorescent changes.

DNA-related probes have the function of recognition and enabled the efficient, selective, and accurate detection of heavy metal ions, such as Hg^2+^, Ag^+^, Pb^2+^, Cd^2+^, and Cu^2+^. The ssDNA with excellent abilities for heavy metal ion recognition is one type of aptamer, and several common schematics of metal ion detection based on aptamer are shown in Figure 2. Hu et al. (2016) developed an ultrasensitive homogeneous fluorometric assay for Hg^2+^ based on gold nanoparticles (AuNPs) and T-rich aptamer with a LOD of 2.5 pM. A novel and simple fluorescent biosensor was realized through the fluorescence quenching of graphite carbon nitride functionalized with ssDNA aptamer for Hg^2+^ detection. The ssDNA aptamer converted to the hairpin-shaped DNA in the presence of Hg^2+^, and this sensor exhibited excellent selectivity and sensitivity with a LOD as low as 0.17 nM (Li et al., 2017). An ultrasensitive approach for the determination of Pb^2+^ by using a specific aptamer and fluorescent perylene with a LOD of 0.1 ng ml^−1^ (Yan et al., 2017). Sun et al. introduced magnetic nanomaterials to aptamers fluorescent sensors to improve the detection performance of Hg^2+^. The presence of Hg^2+^ influenced the combination between the signal transduction probe and aptamers, and the aid of magnetic separation would remove aptamers from the system, resulting in highly accurate fluorescent detection with a LOD of 0.2 nM in river water and ribbon fish (Sun et al., 2018). Hg^2+^ and U^4+^ were detected with C-rich hairpin DNA loaded with silver nanoclusters (AgNCs) as a fluorescent probe, due to the different affinity between hairpin DNA and any of Ag^+^, Hg^2+^, and U^4+^ (Lin et al., 2019).

G-quadruplexes are four guanines forming a square planar tetrad from single-strand state as the presence of metal ions, so G-quadruplexes have been widely employed for the detection of metal ions. Fluorescent sensors and DNA molecules containing G-quadruplexes were designed differently as the fluorescent molecules and nanomaterials changed. Nine dual-fluorophore labeled DNA probes containing G-quadruplexes were prepared to select the sensitive probe for Tl^+^ detection. DNA adsorption by AuNPs was inhibited by Tl^+^ due to DNA folding, resulting in color changes upon salt addition and a LOD of 4.6 μM for Tl^+^ (Hoang et al., 2016). A multi-target biosensor based on K^+^-induced fluorescent G-quadruplex and N-methyl mesoporphyrin IX could change into a more stably non-fluorescent G-quadruplex structure as adding Pb^2+^ or Hg^2+^. The fluorescence decreased as the DNA structure changed, allowing detection of Pb^2+^ and Hg^2+^ with a LOD of 5 and 18.6 nM, respectively (Zhu et al., 2018). The simultaneous detection of Pb^2+^ and Ag^+^ in food and water samples was developed using single-labeled fluorescent DNA sensor. The sensor could combine with Pb^2+^ and Ag^+^ to form G-quadruplex, with a LOD of 96 and 21 pM, respectively (Zhang et al., 2018). Two hairpin DNA probes containing the G-quadruplex sequence as signal amplification elements and thioflavin T as fluorophore were used for Hg^2+^ detection with a LOD of 10.2 pM (Hen et al., 2017). In the Hg^2+^ sensor, one of two hairpin sensors were influenced by the presence of Hg^2+^, and then cross-opening of another hairpin probe was triggered based on the strand displacement principle, while thioflavin T bounded to the new G-quadruplex structure leading to the obvious fluorescence enhancement.

DNAzymes can bind with certain ions due to exceptional recognition abilities, and the heavy metal ions have significant impact on efficient catalytic activities of DNAzymes. The activities of DNAzymes are activated by the addition of relevant target ions, such as Pb^2+^, Cu^2+^, Zn^2+^, Mn^2+^, Co^2+^, Ni^2+^, and Hg^2+^, leading to DNA amplification or DNA cleavage from the specific positions. Various combination strategies of DNAzymes and fluorescent materials have been used for heavy metal ions detection (McGhee et al., 2017; Yun et al., 2017; Chen et al., 2020c; Li et al., 2020c; Ren et al., 2020). Zn^2+^-dependent cleavage DNAzyme was used in the ultrasensitive fluorescent quantitative biosensor for its sensitive and specific recognition of Zn^2+^. The DNA substrate chains produced a strong fluorescent signal owing to large amounts of double-stranded DNA through PCR amplification. Cleavage of DNAzyme was activated in the presence of Zn^2+^, leading to the substrate chains cleavage by 17E at the proper temperature, and the amplification reactions of cleaved substrate chains stopped, resulting in the decrease of fluorescent signal. This quantitative detection system for Zn^2+^ was established with a LOD of 58.61 pM (Li et al., 2019). Li and co-workers established a novel Hg^2+^ detection system with the terminal deoxynucleotidyl transferase (TdT), fluorescent signal probes, SiO_2_ microspheres with a capture DNA 1, and reporter AuNPs with capture DNA 2. Coupled with the target Hg^2+^-induced strand displacement amplification, dendritically amplified fluorescent signal probes were assembled onto SiO_2_ microspheres by multi TdT-based DNA extension reactions and bio-barcode reaction, revealing extraordinary sensitivity for Hg^2+^ assay with a LOD down to 1.0 aM (Li et al., 2020b).

## Challenges and Outlook

Different types of fluorescent materials applied for heavy metal ions detection have been summarized in this mini review. Fluorescent molecules and nanomaterials have unique properties and can significantly improve the sensitivity through rationally designing the structure and innovating synthesis methods. DNA-related sensors are attractive and guarantee highly selective recognition toward heavy metal ions. The combination of different types of fluorescent materials has enabled the efficient and accurate detection of metal ions in real samples for biosening and bioimagining.

Fluorescent materials have been sucessfully used for heavy metal ions detection in real samples of complex matrices, such as water, human body fluids, food, and soil, and can be applied for bioimagining to show the distribution of heavy metals. The sample types, selectivity, and sensitivity of most researches based on fluorescent materials can satisfy the metal ions detection demand of evidence analysis in forensic chemistry field. Despite the great progress achieved in these areas, there are still challenges that need to be addressed. First, the number of targeting different metal ions is still quite limited. Further work is needed to establish more kinds of metal ions detection assays with different emission wavelengths to alllow parallel on-site tests. At the same time, it is rather important to develop more fluorescent marterials to detect and quantify different metal ions in a single sample simultaneously. However, not all the fluorescent marterials are suitable for the heavy metal ions detection in forensic chemistry field. A schif base sensor was developed to recognize three specific metal ions (Ni^2+^, Fe^3+^, and Mg^2+^) equally and also could obtain effcient regeneration (Iyappan et al., 2020). The sensor meets all the requirements to be an excellent fluorescent method for wide applications in the field of biosensing and imaging, and the response ability to multiple metal ions and regeneration are both acceptable and required from the perspective of forensic chemistry, but the sensor is not suitable for forensic chemistry. With the disciplinary nature of forensic chemistry, it is more acceptable that different ions can be confirmed separately with different signals. Moreover, it is a rather difficult task to develop a standard or simple procedure to convert different evidences of complex matrices in the crime scene to the acceptable samples of the methods metioned above, and there are few articles focusing on sample preparation. To address these challenges, it is desired to introduce new recognizing materials to the current system or fabricate new fluorescent materials with better fluorescence stability, multi-recognition, and fluorescence quantum yield, as well as application for more real samples.

Along with the tremendous progress in the field heavy metal ions biosensing and bioimagining, it is expected that fluorescent materials-based methods will bring about profound promising changes in forensic chemistry, play essential roles in practical applications for heavy metal ions detection, and improve the efficiency and intensity of crime prevention and solution.

## Author Contributions

All authors listed have made a substantial, direct and intellectual contribution to the work, and approved it for publication.

## Conflict of Interest

The authors declare that the research was conducted in the absence of any commercial or financial relationships that could be construed as a potential conflict of interest.
